# Nutritional importance and industrial uses of pomegranate peel: A critical review

**DOI:** 10.1002/fsn3.3320

**Published:** 2023-03-23

**Authors:** Huma Bader Ul Ain, Tabussam Tufail, Shahid Bashir, Nabia Ijaz, Muzzamal Hussain, Ali Ikram, Muhammad Adil Farooq, Shamaail A. Saewan

**Affiliations:** ^1^ University Institute of Diet and Nutritional Sciences, Faculty of Allied Health Sciences The University of Lahore Lahore Pakistan; ^2^ University Institute of Food Science and Technology, Faculty of Allied Health Sciences The University of Lahore Lahore Pakistan; ^3^ Department of Food Sciences Government College University Faisalabad Faisalabad Pakistan; ^4^ Department of Food Science and Technology Khwaja Fareed University of Engineering and Information Technology Rahimyar Khan Pakistan; ^5^ Department of Food Sciences College of Agriculture, University of Basrah Basrah Iraq

**Keywords:** animal feed, antioxidants, bioactive compounds, functional properties, human health, industrial use, nutritional composition, pomegranate peel, punicic acid

## Abstract

Pomegranate (*Punica granatum* L.), commonly known as a seeded or granular apple, is a delectable fruit eaten worldwide. Pomegranate is one of the healthiest fruits, with a high concentration of phenolic compounds. Large quantities of byproducts, such as seeds and peels, are produced during the pomegranate juice extraction process, which causes disposal problems and environmental contamination. Pomegranate peel (PoP), which accounts for around 30%–40% of the fruit component, is a byproduct of the fruit juice manufacturing industry. PoP is a rich source of polyphenols including phenolic acids, tannins, and flavonoids, especially anthocyanin. These peels offer several functional and nutraceutical qualities owing to their bioactive ingredients, including lowering blood pressure, reducing oxidative stress, lowering cholesterol levels, and restoring heart health. PoPs have a variety of biological effects, including the ability to resist pathogenic microbes effectively, and used as an additive in various food applications. The current review focuses on the PoP's nutritional and practical attributes, as well as their functions as food additives and functional food preparations.

## Graphical abstract

The current review focuses on the pomegranate peel's nutritional and practical attributes, as well as their functions as food additives and functional food preparations.

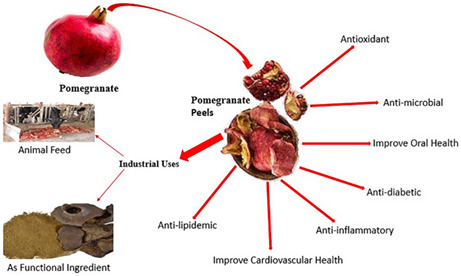

## 1 INTRODUCTION

Annually, the United States dumps approximately 31% (almost 133 billion pounds) of its food supply (Lins et al., 2021). About 61.2 billion pounds of food waste are in landfills. Not only is this excessive waste harmful to the environment, but it is also a significant contributor to climate change problems, as food waste accounts for 18% of all methane emissions from landfills in the United States. Even more food waste and methane emissions are 30%–40% from fruit and vegetables in the trash (Bayram et al., 2021). As one of the biggest businesses in the world, the food industry is crucial to numerous economies. In any case, the quick development of the world's population and the requests of the food supply chain will lead to a quick increase in nourishment generation within 50 years. Agreeing to a report by the Food and Agriculture Organization of its member countries (FAO, 2011), almost one‐third of the food for human utilization is misplaced or squandered around the world (Despoudi et al., 2021).

Food scraps comprise materials proposed for human utilization, which are then taken out, lost, disintegrated, or tainted. The issue of food squandering is getting increasingly dire and influences all spaces of waste administration from assortment to removal. Like retailers and end clients, all members of the food inventory network, agribusiness and industry are engaged in the quest for feasible arrangements (Girotto et al., 2015). Food waste is tormented by environmental factors (along with weather alternatives and air, water, and land pollution) and social factors (which include the aforementioned populace boom and new intake trends) (Garcia‐Garcia et al., 2015). The loss and waste of fruits and vegetables are not only related to food waste but also indirectly include the loss of essential resources (such as land, water, fertilizers, chemicals, energy, and labor). They generate most environmental waste (Sagar et al., 2018).

A large amount of fruit and vegetable waste can be recycled in various ways, including immediately discarding it in a landfill, drying to a stable state (humidity 10%) for off‐season feed, and processing by biotechnology to produce superficial chemical peels. Vinegar, citrus extract and acidic corrosive are gotten from side‐effects of the leafy foods industry. Squander from potato and wheat starch‐preparing plants can be matured into ethanol (Khedkar & Singh, 2014). When handling foods grown from the ground, many wastes like skins, seeds, fluids, and molasses are created. These squander are a decent wellspring of sugars, proteins, fibers, nutrients, and minerals. The waste can be used to make natural blossoms through maturation (Kaur et al., 2019). The utilization of leafy food squanders, mainly shrubs, in the improvement of significant worth‐added items are harmless to the ecosystem approach to set out new organizations open doors and highlight, and economic courses in organizations that produce valuable waste (Kumar et al., 2020).

Phenolic contents in the strip of lemon, orange and grapefruit are 15% lower than the all‐out phenol in the strip. Lemon and lime strip oils are generally used as flavor enhancers in delicate and alcoholic food varieties and fluids (Gorinstein et al., 2001).

**FIGURE 1:**
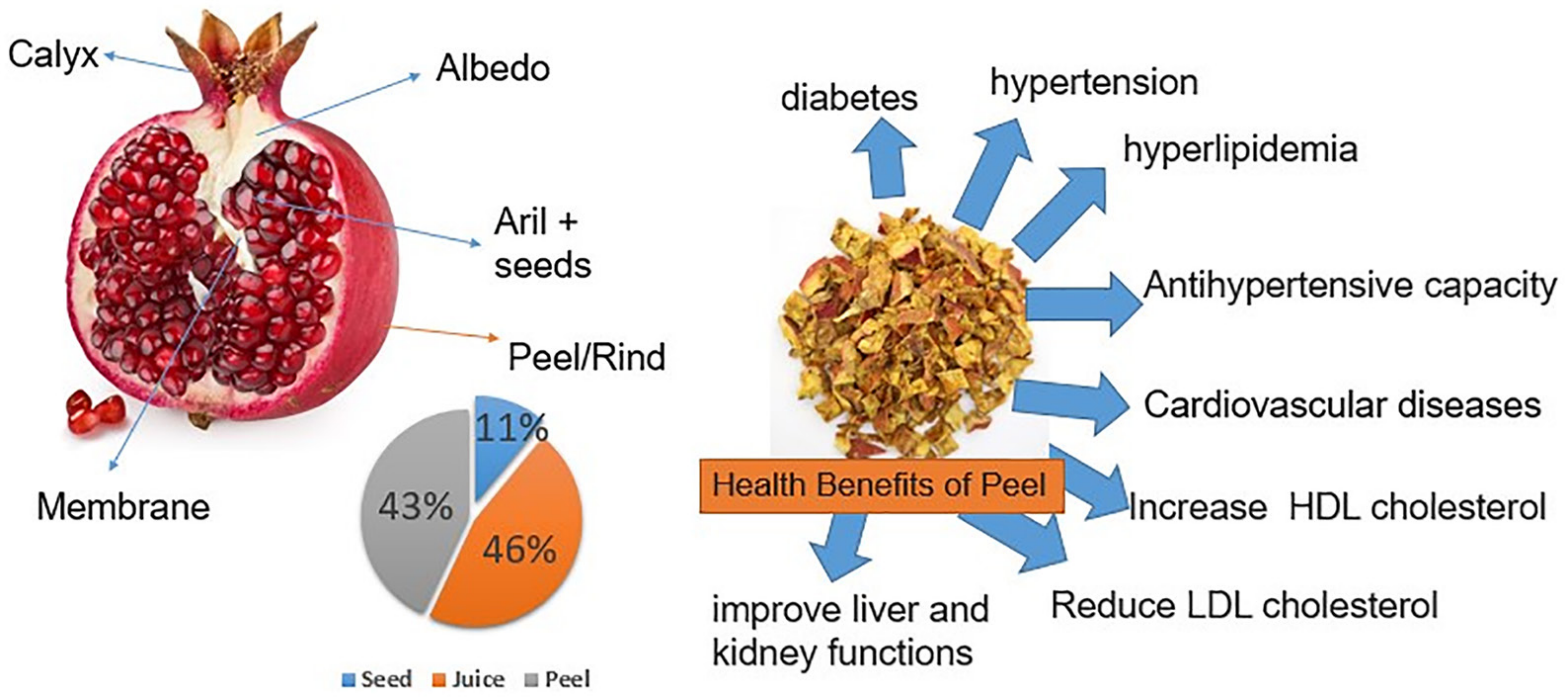
Parts of pomegranate fruit and its therapeutic effects.

Pomegranate (*Punica granatum*) is a small tree native to the Middle East and is now found in the Mediterranean, China, India, South Africa, and the United States. In the past decade, the prevalence of pomegranate has increased significantly, especially after it has been proven to have antimicrobial and antiviral properties. It has anti‐cancer properties, powerful antioxidants and resistance to mutations. The pomegranate fruit consists of three parts: seed, juice, and peel (Figure 1). In particular, peeling is traditionally used as a natural remedy for infectious diseases (Elfalleh et al., 2012; Kharchoufi et al., 2018).

### 1.1 Pomegranate peel

**FIGURE 2:**
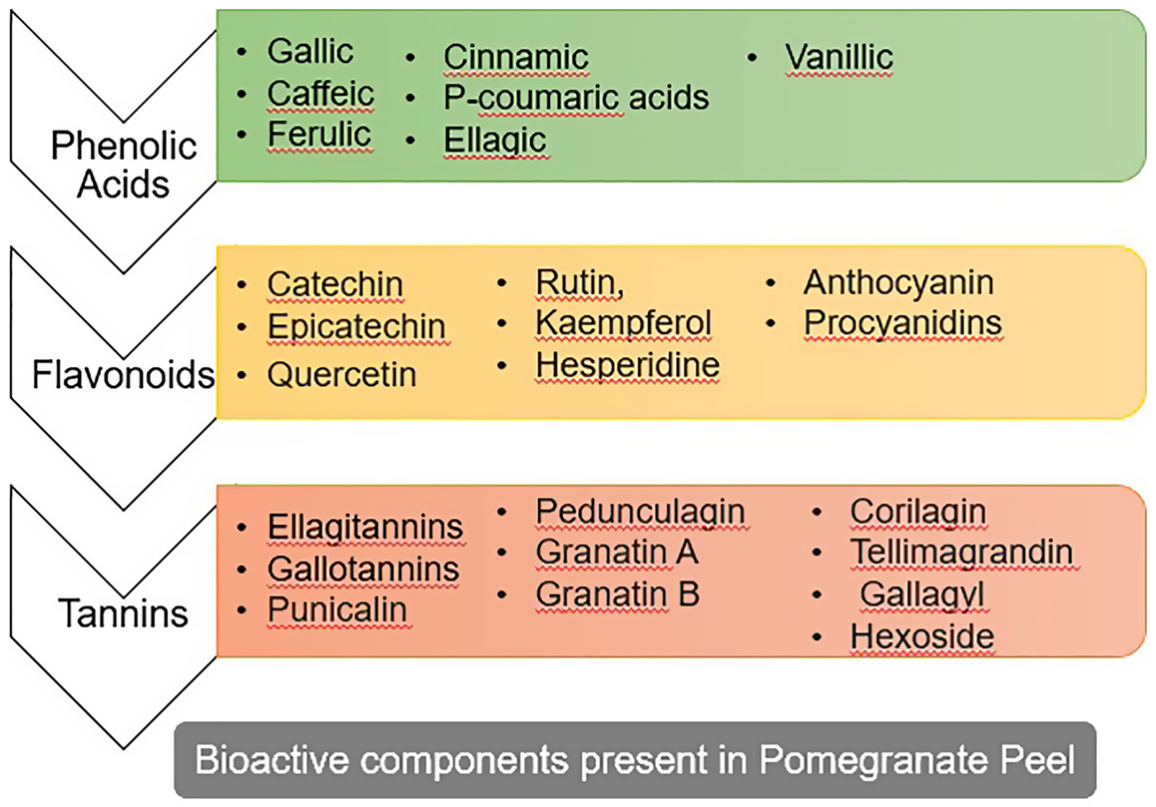
Bioactive components present in the pomegranate fruit peel.

Many researchers have affirmed that pomegranate peel (PoP) is a rich wellspring of bioactive mixtures, including ellagitannins, catechins, rutin, and epicatechins (Figure 2). Previously, pomegranate was broadly used in daily medication to destroy parasites and to treat and fix ulcers, wounds, acidosis, loose bowels, microbial contaminations, and respiratory illnesses. PoP has higher cancer prevention agent movement than heart and juice (Mphahlele et al., 2016). Pomegranate skin accounts for 40% of the whole fruits, and its antioxidant activity is higher than that of the edible (Abid et al., 2017).

The peel and seeds of pomegranate account for about 54% of the fruit and the pomegranate were lost after pressing. Pomegranate is composed of 43% of bark, 11% of seeds, and 46% of juice (Ko et al., 2021). In particular, PoP comprises nearly 30%–40% of the fruit. The skin is rich in polyphenols, and because polyphenols are cancer prevention agents, they can show the pharmacological capability of pomegranate (Ijaz et al., 2020). Literature demonstrated that peel contains more bioactive compounds than other parts of the fruit (Akhtar et al., 2015; Rahnemoon et al., 2016; Ullah et al., 2012; Wang et al., 2011).

PoP is a rich wellspring of hydrolyzable tannins in ellagitannins. It tends to be closed from past examinations that the pomegranate strip is wealthy in comparative fixings and has cell reinforcement potential. Dyes are made from PoP. An extract or a powder from the peel of pomegranates, this dyestuff is high in tannin and improves the light and wash fastness of any dye with which it is mixed. PoP yields soft yellows to green yellows when used as a dye (Ajmal et al., 2014). The industry price of PoP is very low. It can be used as an added value for various products such as biscuits, pasta and cakes (Saroj et al., 2020), fresh and concentrated fruit juice, herbal tea, and jam. (Karimi et al., 2017).

### 1.2 Nutritional and chemical composition

Every day, PoPs are used in various food varieties owing to their great nutritional and chemical composition (Kandylis & Kokkinomagoulos, 2020). PoP contains high levels of carbohydrates (59.60%) followed by moisture content (5.40%–5.95%), protein content (4.90%–8.97%), ash content (3.40%–4.22%), fiber content (16.30%–19.41%), and fat content (0.85%–1.26%) (El‐Hamamsy & El‐khamissi, 2020; Khan et al., 2017). The chemical and nutritional composition of PoP were presented in Table 1.

**TABLE 1:** Chemical and nutritional composition of pomegranate peel.

	Moisture	Total sugar/CHO	Reducing sugar	Protein	Ash	Fiber	Pectin	Fat	Total solids	Reference
Pomegranate peel per 100 g	5.40	17.70	–	4.90	3.40	16.30	–	1.26	94.50	(Khan et al., 2017)
Pomegranate peel (%)	67.26%–73.23%	30.65%–34.83%	–	3.96%–7.13%	3.71%–4.97%	28.10%–33.93%	6.8%–10.1%	–	–	(Ko et al., 2021)
Pomegranate peels (%)	6.95	59.60	–	8.97	4.22	19.41	–	0.85	–	(El‐Hamamsy & El‐khamissi, 2020)
Pomegranate peel per 100 g	5.40	17.70	4.34	4.90	3.40	16.30	–	1.26	94.50	(Middha et al., 2013)

### 1.3 Punicic acid in pomegranate peel

Punicic acid, a bioactive compound of pomegranate seed oil, has gained wide attention because of its therapeutic potential. Polyunsaturated fatty acid composition, predominantly comprising of punicic acid, is highly present in pomegranate seed oil. Punicic acid is named after its principal source pomegranate. Among all the sources of punicic acid, it is most abundantly present in pomegranate seed oil. The other sources of punicic acid are snake gourd seed oil. Punicic acid has been proven to protect collagen fibers found in the skin, accelerating wound healing, and reducing the appearance of scars. The anti‐inflammatory property of punicic acid has also effectively addressed skincare ailments like eczema and psoriasis (Shabbir et al., 2017). Punicic acid decreases oxidative damage and inflammation by increasing the expression of peroxisome proliferator‐activated receptors. Punicic acid is an important nutraceutical compound in the prevention and treatment of neurodegenerative diseases such as Alzheimer's, Parkinson's, and Huntington's disease (Guerra‐Vázquez et al., 2022).

DPPH assay is the most adopted procedure to assess the antioxidant capacity of corresponding samples mainly due to its short run time, simplicity, and stability during the whole experimental process. Principally, the antioxidant potential of respective samples is analyzed depending on their potential to reduce DPPH• by donating hydrogen atom that can also be authenticated spectrophotometrically due to the loss of dark violet color of the tested solution. The results help in inferring that PoP antioxidant extracts retard food deterioration which mainly initiates due to the production of free radicals. Punicalagin content in PoP extracts revealed significantly strong antioxidative capacity when evaluated using DPPH assay, validating its therapeutic role (Khalil et al., 2017).

Pomegranate juice was used in human and rat diet against atherogenicity and atherosclerosis, respectively. Findings of the investigation concluded that pomegranate juice has the potential to combat these disorders owing to its antioxidative properties (Aviram et al., 2000).

### 1.4 Antioxidant activity of pomegranate peel

Pomegranate has become one of the foods richest in polyphenols. It has a high antioxidant capacity and health benefits, so it is very popular, which is why it is often called “super fruit.” Because of the great substance of phenolic acids, flavonoids, and other polyphenol compounds, pomegranate can be used as a successful safeguard of different dynamic oxygen. As such, ordinarily happening phenolic mixtures can restrain food oxidation and increment the timeframe of realistic usability and nature of food. Moreover, the food business desires to supplant synthetic substances with regular mixes to improve food handling (Derakhshan et al., 2018). The presence of mixtures with antioxidant characteristics in PoP is a vital wellspring of more dynamic cell reinforcements (Singh et al., 2002).

Cell reinforcements are compounds added to food varieties, particularly lipids and lipid‐containing frameworks. They can broaden the timeframe of realistic usability of food sources by hindering the lipid peroxidation measure instead of simply making substances harm food sources. However, they additionally produce free extremists, for example, peroxy‐free revolutionaries and hydroxyl‐free revolutionaries, which are believed to be engaged in carcinogenesis, mutagenesis, and maturing (Yasoubi et al., 2007).

A few examinations have shown the antioxidant characteristics of the side effects of pomegranate extract. PoP removal is applied to mackerel to repress the lipid oxidation of fish unsaturated fats. The cell reinforcement impact of the concentrate was analyzed with the antioxidant agent impact; butylated hydroxyl toluene, an incredibly engineered cancer prevention agent broadly used in the food business (Andrade et al., 2019).

Natural antioxidants are better than synthetic antioxidants because of their lower activity; the amount of natural antioxidants added is much larger than that of active antioxidants. However, the simple action depends on the particular conditions and food arrangement (Pokorný, 2007).

Contrasted with different concentrates, the methanol removed in the pomegranate strip has the most elevated cell reinforcement action and phenol content (Tehranifar et al., 2011). 2,2‐Diphenyl‐1‐picylhydrazine (yellow) is a stable free radical that can accept electrons or hydrogen‐free radicals from antioxidants. In this study, the inhibitory activity of PoP extracts on DPPH free radicals is very different. It showed the highest antioxidant activity in all extracts, so it was chosen to test its effects on lipid peroxidation, hydroxyl radical scavenging activity and human low‐density lipoprotein oxidation (Malviya et al., 2014).

### 1.5 Flavonoids in pomegranate peel

Pomegranate is made from different flavonoids, representing 0.2%–1.0% of the organic product. About 30% of all anthocyanins contained in pomegranate are in the peel (Omer et al., 2019). The flavonoid content in PoP is about 12.4 times higher than in juice and seed (Kalaycıoğlu & Erim, 2017).

Flavonoids (mainly anthocyanins) determine the color of pomegranate fruit, but the content of tannins in the whole pomegranate skin is also higher than the total flavonoids (Singh et al., 2018).

PoP may be a potential supply of flavonoids, equivalent to catechins, epicatechins, quercetin, anthocyanins, and procyanidins. The composition of flavonoids varies with the fetus's biological process stage, and the concentration within the PoP varies with the sort of pomegranate. The compound and rutin are thought to be the very best content of flavonoids in contemporary dishes from Granada (Mphahlele et al., 2017). The composition of polyphenols and total phenolic content of PoP extract was shown in Table 2.

**TABLE 2:** Composition of polyphenol compounds and total phenol of pomegranate peel extract.

Polyphenolic compounds (mg/g)	Source: Ibrahium (2010)	Source: Gullón et al. (2020)
Punicalagin	296	–
Delphinidin	34	–
Cinnamic acid	42	2.5
Punicalin	15	–
Pelargonidin	21	–
Coumaric acid	32	0.91
Ellagic acid	18	12.56
Quercetin	40	–
Ferulic acid	28	–
Gallic acid	71	2.5
Kaempherol	62	–
Sinapic acid	17	–
Cyanidin	26	–
Luteolin	33	–
Caffeic acid	11	–
Chlorogenic acid	–	1.56
Total phenolic compounds	867 mg/g	mg/g

## 2 ETHNOMEDICINAL USES OF POMEGRANATE PEEL

### 2.1 Type II diabetes

Diabetes is possibly the main pandemic‐persistent metabolic sickness on the planet (Middha et al., 2014). Diabetes is sometimes characterized by hyperglycemia that ends up in disorders of carbohydrate, lipid, and macromolecule metabolism (Barathikannan et al., 2016). The antioxidant‐rich PoPs and seeds have antecedently shown protection against diseases regarding aerophilous stress, together with diabetes (Manna et al., 2019).

The anti‐diabetic impact of the active ingredient of PoP is closely related to the inhibition of α‐glucosidase and the increase of aldohexose absorption. The active ingredients in PoP have also been shown to cut back polygenic disorder‐related cardiopathy by inhibiting beta‐lipoprotein oxidation. The acetate fraction (F5) within the crude alcohol extract of PoP has innovative inhibitor and anti‐diabetic activities that are equivalent/better than the quality used. It is used as a nutritionary health product for the interference and treatment of kind a pair of diabetes and its connected complications (Arun et al., 2017).

### 2.2 Alzheimer's disease

Alzheimer's disease might be a reformist neurodegenerative problem and hence the most overarching assortment of derangements described by a reformist decrease in memory, conduct, and mental component capacities inside the matured populace. It influences people and has become a critical clinical and social weight in non‐industrial nations (Subash et al., 2014). It is accepted that Alzheimer's is a complex multifactorial sickness, and there are no successful restorative specialists to moderate or forestall the improvement of the infection (Braidy et al., 2016).

Alzheimer's sickness might be a persistent illness that has not been treated until late. Momentum treatment strategies downsize sickness movement, are costly, rarely cause aspect impacts, and are poorly designed for the patients (Morzelle et al., 2016). In Alzheimer's infection, oxidative pressure is fundamental (Harakeh et al., 2020).

The concentrates from the shell showed more significant levels of phenols related to cell reinforcements, which is why they were picked as likely inhibitors of acetylcholinesterase, which might be a key impetus worried inside the advancement of Alzheimer's disease. The engineered sap compounds in the pomegranate strip show a harsh effect on acetylcholinesterase, which relies on the grouping of phenol (Morzelle et al., 2019).

### 2.3 Oral cavities

Get the first profit from PoP (pomegranate) because agricultural waste is tooth glue framed from PoP squander, financial and ecological insurance (Abbas, 2014). PoP is a rich wellspring of polyphenols, including penicillin, punicalagin, and ellagic corrosive, yet it is viewed as rural waste. Reports indicate that pomegranate‐determined items have various medical advantages, including antibacterial properties (Smith et al., 2020).

Pomegranate has significant utilization in the field of dental well‐being. Clinical reports have affirmed that this current cancer prevention agent is dissolving the underlying driver of tooth rot with its force at the biochemical level (Umar et al., 2016). PoP extract is a promising strategy for the treatment of oral thrush. Because of its viability, oral thrush tends to be used as a characteristic substitute (Bassiri‐Jahromi, 2018a, 2018b). Pomegranate can hinder Streptococcus mutants because of its tannic corrosive antimicrobial properties so it could be a potential enemy of caries specialists. It can keep different microorganisms from clinging to the oral hole (Menezes et al., 2006; Prasad & Kunnaiah, 2014).

### 2.4 Human cancer cells

As indicated by reports, the work of PoP has been hostile to tumor impacts, along with the annihilation of tumor cell expansion, cell cycle, intrusion and angiogenesis. Pomegranate strip separation has appeared to moderate cell expansion in different neoplastic cell lines. Pomegranate strip affects the extension of carcinoma cells of arranged subatomic subtypes (Bassiri‐Jahromi, 2018a, 2018b).

The counter‐proliferative properties of pomegranate strip extricate are outlined in shifted human malignancy cells (human non‐little cell carcinoma A549, human PC‐3 glandular disease cells, SKOV3 testicle malignant growth cells and SKOV3 malignant growth cells). Carcinoma (MCF‐7) it has been discovered that MCF‐7 bosom adenocarcinoma cells are the preeminent touchy (Vučić et al., 2019).

The counter‐estrogen movement of polyphenols and flavonoids in pomegranate strips can meddle with aromatase action by disturbing estrogen blend, which may be found in cells with six chemical receptors (e.g., MCF‐7 and SKOV3). Its capacities as a development factor. Although SKOV3 female inside regenerative organ malignancy is not estrogen‐reliant, as opposed to elective steroid‐and androgen‐touchy disease cells, these cells are less delicate to the antiproliferative movement of pomegranate strip removal. Furthermore, the counter proliferative effect of pomegranate strip separate seems, by all accounts, to be tumor‐explicit because chemical ward malignancy cells are horrendously touchy to the counter‐tumor impacts of those concentrates (Modaeinama et al., 2015).

## 3 INDUSTRIAL USES OF POMEGRANATE PEEL

It is possible to take advantage of pomegranate byproducts, as they are a rich source of bioactive compounds such as flavonoids, antioxidants, phenols, polyphenols, and anthocyanins. The peels are packed with powerful antioxidants that help fight against dangerous and life‐threatening heart diseases. PoP lowers cholesterol levels, reduces oxidative stress, restores heart health, and lowers blood pressure. It is facial scrub and exfoliates while also containing sun‐blocking agents that offer protection from the sun's harmful UV rays. Therefore, there is a need to highlight all the wastes related to the food and food industry so that awareness can be created among the public and their utilization can be improved.

### 3.1 Animal feed

**FIGURE 3:**
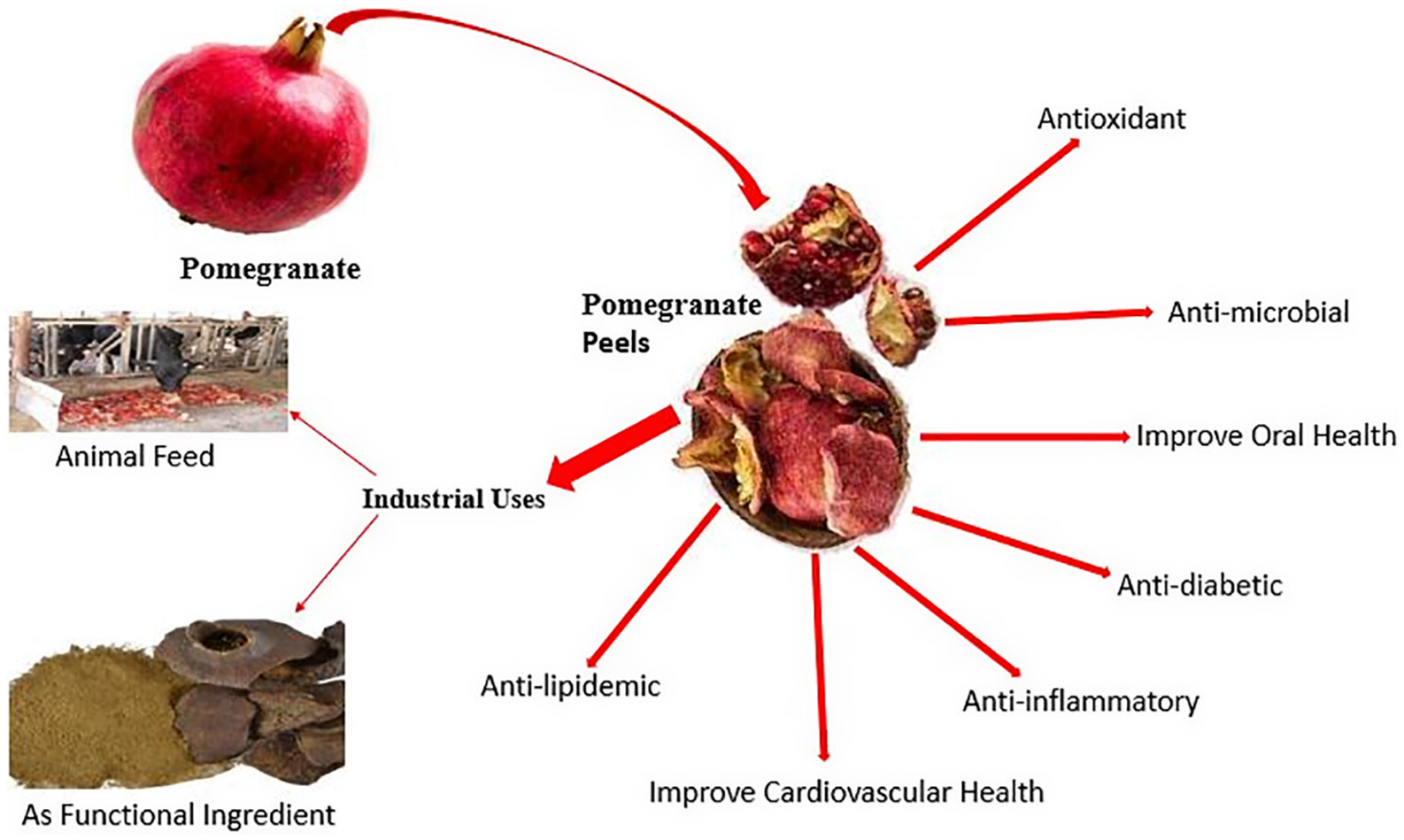
Nutritional and functional properties of pomegranate peels.

PoP is an abundant source of ellagitannins (antioxidants); therefore, it is used to prevent and reduce farm animal diseases and improve meat products, creating it a pretty part of animal feed (Figure 3). Recent studies have additionally shown that increasing the antioxidant content of the beef diet will facilitate the cleaning of the pomegranate. Industrial waste potable can be recycled using tannins; therefore, the remaining byproducts (such as solid waste on the skin) can be produced as feed additives at a low‐cost price. These additives are rich in helpful nutrients (Kushwaha et al., 2013).

Pomegranate byproducts have a high worth as feed. For recent skin, this study clearly shows that consumption positively influences the expansion of calves and the accumulation of R‐tocopherol within the plasma. Nutrients are directly concerned with the inhibitor system. However, this does not mean that adding them to the diet will improve animal health. However, whenever used legally, the bioavailability of minerals, nutrients and polyphenols in new and kept PoP could have a supportive preventive impact (Shabtay et al., 2008).

PoP affects the daily nutrient intake throughout the Karadi Lamb digestion test. Compared with alternative treatments, the typical biological process worth of lambs fed with 1% PoP was increased to the lower phenol content in lambs fed with 1% PoP (Sadq et al., 2016).

### 3.2 Active packaging

Fish gelatin could be a sensible supply of biopolymers for packaging films due to its perishable and good film‐forming properties because of its high content of myofibril. The expansion of PoP powder improves the lastingness of the film, however, because of the insoluble particles. It additionally causes a rise in the water vapor porousness of the film. PoP powder is added to fish gelatin film‐forming solution to develop an active packaging film with potent inhibitor and medicament properties (Hanani et al., 2019). Polyethylene terephthalate with active pomegranate skin film can extend the pork period higher than polypropene with pomegranate skin film and may exceed the shelf life of pork (Hu et al., 2017).

The extract of PoP is supplementary to zein to create a vigorous package, thereby raising the valuable properties of the film. Once packaging Kalari cheese, the film will significantly curtail the oxidization reaction and microorganism deterioration throughout storage. It is used as a food safety antimicrobial and inhibitor packaging material within the cheese industry. In addition, the film does not affect the beneficial bacterium of the cheese, thereby increasing its popularity (Mushtaq et al., 2018). Microbiological perceptions demonstrate the probability of mistreating pomegranate polyphenols in zein dynamic layers as common additives. Moreover, cold atmospheric plasma treatment improved the stretching at break and strength of the zein‐based nanocomposite film to change the layer surface. Mistreatment of pomegranate strips may likewise settle the garbage removal disadvantage (Cui et al., 2020).

Mung bean protein‐based food film has been built up with various groupings of pomegranate strips. The convergence of the pomegranate strip will build the thickness, contact point and fume porousness of the mung bean layer, decreasing its water and dissolvability. As an economic side effect of the food business, it has the valuable capability of being brought into the mung bean protein film to upgrade its deliberate organic properties (Moghadam et al., 2020).

Moreover, Kumar et al. (2021a, 2021b) used PoP extract (0.2 g/mL) as an active antioxidant agent for the preparation of chitosan–pullulan (50:50) composite edible coating and probed its effect on the shelf life of mango fruit during 18 days of storage period at 23°C and 4°C. This has resulted in the improvement of mango fruit shelf life.

### 3.3 Yogurt

Pomegranate is considered a good source of antioxidants and antibacterial drug activity. Some researchers used PoPs in hard milk; however, tannins restrained the expansion of bacteria. Adding 0.5% PoP to organic stirred yogurt will improve overall tolerance, consistency, and texture. Because of the rise of protein, dry matter, and cellulose in PoP and addition of pomegranate to organic cooked yogurt exceeds the period of organic fried yogurt. It improves the 21‐day survival rate of probiotic culture (Ibrahim et al., 2020). Expanding the extent of PoP extricate brought about a very diminishing viciousness of the blended yogurt. The thickness of the examples containing 20% and 25% PoP removal was almost similar (El‐Said et al., 2014).

In a study, Kennas et al. (2020) elaborated that yogurt with PoP and nectar is prepared and freeze‐dried. The outcomes show that freeze‐dried dairy item has good physical and compound properties, especially low wetness content, which may broaden its timeframe of realistic usability. Once PoP and nectar are added to yogurt, the phenol substance and inhibitor movement are impressively expanded. In this manner, adding PoP can fulfill the interest in deliberate food varieties.

### 3.4 Meat industry

Owing to the antimicrobial and antifungal properties of PoP can be used as an effective and natural option for synthetic preservative agents to preserve the quality of food during storage (Kandylis & Kokkinomagoulos, 2020; Ullah et al., 2012; Khan & Hanee, 2011; Ibrahium, 2010). As poultry items are prone to lipid and protein oxidation and are perishable, the industry is in constant search of synthetic‐free additives that help in retarding the oxidation process, leading to the development of healthier and shelf‐stable products. PoP extract is a superb variety of artificial antioxidants (Das et al., 2021). PoP extract inhibits the lipid chemical reaction and also the decomposition of meat pigments, serving to prevent rancidity and stabilize the flesh color of meat and meat product and their shelf life (Smaoui et al., 2019) and improve the quality and functional characteristics (Gullón et al., 2020).

PoP extract is a good antioxidant that will enhance the alpha‐tocopherol content of broilers because adding 200 and 300 mg/kg of PoP extract to the diet can increase the antioxidant potential and assess the breast muscle quality of broilers (Saleh et al., 2017).

### 3.5 Cookies

Adding PoP to biscuits will improve the organic process worth of the product. It has been found that adding PoP to flour can extend the period of cookies to realize high inhibitor potential. Similarly, in cookies with additives, the content of calcium, potassium, iron, and atomic number 30 also magnified significantly (Ismail et al., 2014).

In this exploration, a fresh out of the plastic new fixing material made of extra organic product squash was created to typify pomegranate strip removal. Furthermore, the unrefined concentrate showed the best expansion in shading boundaries all through capacity, while the inhibitor movement inside the braced treats was kept at a significant level. All through the entire stockpiling time, the degree of the executives' treatment has been extensively improved. Different treats are frightfully steady regarding oil oxidation (Kaderides et al., 2020).

As a food additive, PoP extract can improve the quality and taste of food and the sensory properties of cookies (Ismail et al., 2016).

### 3.6 Bakery products

The utilization of dried PoP powder improves the dietary and medical advantages of biscuits because the substance of β‐carotene, fiber, and polyphenols in dried PoP powder is higher, which changes the surface, rheology, and tangible properties of the biscuits (Srivastava et al., 2014).

PoP powder is a decent wellspring of naturally dynamic food fixings, yet its monetary worth is little, and it has been used to make biscuits. The expansion of PoP powder brought about a critical expansion in the aggregate and insoluble fiber, Mg, Ca, K, total phenols, and all‐out cancer prevention agent action in the biscuits. The tactile view of biscuits can make PoPs effectively used in utilitarian business cakes, and the mechanical utilization of PoPs can help decrease contamination (Topkaya & Isik, 2019).

## 4 CONCLUSION

Lately, the food business's interest in natural antioxidant agents has consistently expanded, particularly since the amount of adverse toxicological reports of the many artificial compounds has increased. PoPs showed higher antioxidant properties and pose higher in applications for food products. Bioactive substances in PoPs play key roles in anti‐inflammatory, antibacterial, improvement of cardiovascular diseases, anti‐infection, and healing, playing a modulating and interventional role. They can be applied to animal feed to improve feed efficiency. The application of PoP has a promising result as an antioxidant in the food industry. PoP prevents diabetes, especially breast cancer, Alzheimer's disease, and cardiovascular diseases. Punicalagin and ellagic acid in PoP have chemopreventive effects against prostate cancer, breast cancer, and colon cancer, partly associated with the ellagic acid–derived metabolite urolithin. The bioactive substances in PoPs can be used as natural food ingredients to prepare innovative food products. In the case of industry, PoP has shown great results by enhancing food products' nutritional properties, taste, texture, flavor, and shelf life. PoP also has a prebiotic effect. Conclusively, reusing pomegranate byproducts in the fresh food industry could be a sustainable way to reduce environmental impact and costs associated with byproducts disposal, with significant advantages to the product quality and its shelf life followed by improvement in human health.

## CONFLICT OF INTEREST STATEMENT

The authors have declared no conflicts of interest for this article.

## Data Availability

The authors confirm that the data supporting the findings of this study are available within the article.

## References

[fsn33320-bib-0001] Abbas, M. N. (2014). Pomegranate peels: Source of antioxidants extraction and natural dentifrices preparation. European Academic Research, 2(3), 3078–3089.

[fsn33320-bib-0002] Abid, M. , Yaich, H. , Cheikhrouhou, S. , Khemakhem, I. , Bouaziz, M. , Attia, H. , & Ayadi, M. A. (2017). Antioxidant properties and phenolic profile characterization by LC–MS/MS of selected Tunisian pomegranate peels. Journal of Food Science and Technology, 54(9), 2890–2901. 28928529 10.1007/s13197-017-2727-0 PMC5583119

[fsn33320-bib-0003] Ajmal, M. , Adeel, S. , Azeem, M. , Zuber, M. , Akhtar, N. , & Iqbal, N. (2014). Modulation of pomegranate peel colourant characteristics for textile dyeing using high energy radiations. Industrial Crops and Products, 58(1), 188–193.

[fsn33320-bib-0004] Akhtar, S. , Ismail, T. , Fraternale, D. , & Sestili, P. (2015). Pomegranate peel and peel extracts: Chemistry and food features. Food Chemistry, 174, 417–425. 25529700 10.1016/j.foodchem.2014.11.035

[fsn33320-bib-0005] Andrade, M. A. , Lima, V. , Silva, A. S. , Vilarinho, F. , Castilho, M. C. , Khwaldia, K. , & Ramos, F. (2019). Pomegranate and grape byproducts and their active compounds: Are they a valuable source for food applications? Trends in Food Science and Technology, 86, 68–84.

[fsn33320-bib-0006] Arun, K. B. , Jayamurthy, P. , Anusha, C. V. , Mahesh, S. K. , & Nisha, P. (2017). Studies on activity guided fractionation of pomegranate peel extracts and its effect on antidiabetic and cardiovascular protection properties. Journal of Food Processing and Preservation, 41(1), e13108.

[fsn33320-bib-0007] Aviram, M. , Dornfeld, L. , Rosenblat, M. , Volkova, N. , Kaplan, M. , Coleman, R. , Hayek, T. , Presser, D. , & Fuhrman, B. (2000). Pomegranate juice consumption reduces oxidative stress, atherogenic modifications to LDL, and platelet aggregation: Studies in humans and in atherosclerotic apolipoprotein E–deficient mice. The American Journal of Clinical Nutrition, 71(5), 1062–1076. 10799367 10.1093/ajcn/71.5.1062

[fsn33320-bib-0008] Barathikannan, K. , Venkatadri, B. , Khusro, A. , Al‐Dhabi, N. A. , Agastian, P. , Arasu, M. V. , Choi, H. S. , & Kim, Y. O. (2016). Chemical analysis of *Punica granatum* fruit peel and its in vitro and in vivo biological properties. BMC Complementary and Alternative Medicine, 16(1), 1. 27476116 10.1186/s12906-016-1237-3 PMC4967515

[fsn33320-bib-0010] Bassiri‐Jahromi, S. (2018a). In vivo comparative evaluation of the pomegranate (*Punica granatum*) peel extract as an alternative agent to nystatin against oral candidiasis. Iranian Journal of Medical Sciences, 43(3), 296–304. 29892147 PMC5993896

[fsn33320-bib-0011] Bassiri‐Jahromi, S. (2018b). *Punica granatum* (pomegranate) activity in health promotion and cancer prevention. Oncology Reviews, 12(1), 345. 29441150 10.4081/oncol.2018.345 PMC5806496

[fsn33320-bib-0012] Bayram, B. , Ozkan, G. , Kostka, T. , Capanoglu, E. , & Esatbeyonglu, T. (2021). Valorization and application of fruit and vegetable wastes and byproducts for food packaging materials. Molecules, 26(13), 4031. 34279371 10.3390/molecules26134031 PMC8271709

[fsn33320-bib-0013] Braidy, N. , Essa, M. M. , Poljak, A. , Selvaraju, S. , Al‐Adawi, S. , Manivasagm, T. , Thenmozhi, A. J. , Ooi, L. , Sachdev, P. , & Guillemin, G. J. (2016). Consumption of pomegranates improves synaptic function in a transgenic mice model of Alzheimer's disease. Oncotarget, 7(40), 64589–64604. 27486879 10.18632/oncotarget.10905 PMC5323101

[fsn33320-bib-0014] Cui, H. , Surendhiran, D. , Li, C. , & Lin, L. (2020). Biodegradable zein active film containing chitosan nanoparticle encapsulated with pomegranate peel extract for food packaging. Food Packaging and Shelf Life, 24, 100511.

[fsn33320-bib-0015] Das, A. K. , Nanda, P. K. , Chowdhury, N. R. , Dandapat, P. , Gagaoua, M. , Chauhan, P. , Pateiro, M. , & Lorenzo, J. M. (2021). Application of pomegranate byproducts in muscle foods: Oxidative indices, colour stability, shelf life and health benefits. Molecules, 26(2), 467. 33477314 10.3390/molecules26020467 PMC7830841

[fsn33320-bib-0016] Derakhshan, Z. , Ferrante, M. , Tadi, M. , Ansari, F. , Heydari, A. , Hosseini, M. S. , Conti, G. O. , & Sadrabad, E. K. (2018). Antioxidant activity and total phenolic content of ethanolic extract of pomegranate peels, juice and seeds. Food and Chemical Toxicology, 114, 108–111. 29448088 10.1016/j.fct.2018.02.023

[fsn33320-bib-0017] Despoudi, S. , Bucatariu, C. , Otles, S. , & Kartal, C. (2021). Food waste management, valorization, and sustainability in the food industry. Food Waste Recovery, 1, 3–19.

[fsn33320-bib-0019] Elfalleh, W. , Hannachi, H. , Tlili, N. , Yahia, Y. , Nasri, N. , & Ferchichi, A. (2012). Total phenolic contents and antioxidant activities of pomegranate peel, seed, leaf and flower. Journal of Medicinal Plants Research, 6(32), 4724–4730.

[fsn33320-bib-0021] El‐Hamamsy, S. M. , & El‐khamissi, H. A. (2020). Phytochemicals, antioxidant activity and identification of phenolic compounds by HPLC of pomegranate (*Punica granatum* L.) Peel extracts. Journal of Agricultural Chemistry and Biotechnology, 11(4), 79–84.

[fsn33320-bib-0022] El‐Said, M. M. , Haggag, H. F. , El‐Din, H. M. , Gad, A. S. , & Farahat, A. M. (2014). Antioxidant activities and physical properties of stirred yoghurt fortified with pomegranate peel extracts. Annals of Agricultural Sciences, 59(2), 207–212.

[fsn33320-bib-0023] Food and Agriculture Organization of the United Nations (FAO) (2011). Climatic Risk Analysis in Conservation Agriculture in Varied Biophysical and Socio‐economic Settings of Southern Africa, Network Paper 03. Regional Emergency Office for Southern Africa.

[fsn33320-bib-0024] Garcia‐Garcia, G. , Woolley, E. , & Rahimifard, S. (2015). A framework for a more efficient approach to food waste management. International Journal of Food Engineering, 1(1), 65–72.

[fsn33320-bib-0025] Girotto, F. , Alibardi, L. , & Cossu, R. (2015). Food waste generation and industrial uses: A review. Waste Management, 45, 32–41. 26130171 10.1016/j.wasman.2015.06.008

[fsn33320-bib-0026] Gorinstein, S. , Martín‐Belloso, O. , Park, Y. S. , Haruenkit, R. , Lojek, A. , Ĉíž, M. , Caspi, A. , Libman, I. , & Trakhtenberg, S. (2001). Comparison of some biochemical characteristics of different citrus fruits. Food Chemistry, 74(3), 309–315.

[fsn33320-bib-0027] Guerra‐Vázquez, C. M. , Martínez‐Ávila, M. , Guajardo‐Flores, D. , & Antunes‐Ricardo, M. (2022). Punicic acid and its role in the prevention of neurological disorders: a review. Foods, 11(3), 252. 35159404 10.3390/foods11030252 PMC8834450

[fsn33320-bib-0028] Gullón, P. , Astray, G. , Gullón, B. , Tomasevic, I. , & Lorenzo, J. M. (2020). Pomegranate peel as suitable source of high‐added value bioactives: Tailored functionalized meat products. Molecules, 25(12), 2859. 32575814 10.3390/molecules25122859 PMC7355679

[fsn33320-bib-0029] Hanani, Z. N. , Yee, F. C. , & Nor‐Khaizura, M. A. (2019). Effect of pomegranate (*Punica granatum* L.) peel powder on the antioxidant and antimicrobial properties of fish gelatin films as active packaging. Food Hydrocolloids, 89, 253–259.

[fsn33320-bib-0030] Harakeh, S. , Ramadan, W. S. , Al Muhayawi, M. S. , Al Jaouni, S. , Mousa, S. , & Hakeem, K. R. (2020). Pomegranate peel extract lessens histopathologic changes and restores antioxidant homeostasis in the hippocampus of rats with aluminium chloride‐induced Alzheimer's disease. Asian Pacific Journal of Tropical Medicine, 13(10), 456.

[fsn33320-bib-0031] Hu, S. , Wang, H. , Han, W. , Ma, Y. , Shao, Z. , & Li, L. (2017). Development of double‐layer active films containing pomegranate peel extract for the application of pork packaging. Journal of Food Process Engineering, 40(2), e12388.

[fsn33320-bib-0032] Ibrahim, A. , Awad, S. , & El‐Sayed, M. (2020). Impact of pomegranate peel as prebiotic in bio‐yoghurt. British Food Journal, 122(9), 2911–2926.

[fsn33320-bib-0033] Ibrahium, M. I. (2010). Efficiency of pomegranate peel extract as antimicrobial, antioxidant and protective agents. World Journal of Agricultural Sciences, 6(4), 338–344.

[fsn33320-bib-0034] Ismail, T. , Akhtar, S. , Riaz, M. , Hameed, A. , Afzal, K. , & Sattar Sheikh, A. (2016). Oxidative and microbial stability of pomegranate peel extracts and bagasse supplemented cookies. Journal of Food Quality, 39(6), 658–668.

[fsn33320-bib-0035] Ismail, T. , Akhtar, S. , Riaz, M. , & Ismail, A. (2014). Effect of pomegranate peel supplementation on nutritional, organoleptic and stability properties of cookies. International Journal of Food Sciences and Nutrition, 65(6), 661–6, 666. 24725173 10.3109/09637486.2014.908170

[fsn33320-bib-0036] Kaderides, K. , Mourtzinos, I. , & Goula, A. M. (2020). Stability of pomegranate peel polyphenols encapsulated in orange juice industry byproduct and their incorporation in cookies. Food Chemistry, 310, 125849. 31753686 10.1016/j.foodchem.2019.125849

[fsn33320-bib-0037] Kalaycıoğlu, Z. , & Erim, F. B. (2017). Total phenolic contents, antioxidant activities, and bioactive ingredients of juices from pomegranate cultivars worldwide. Food Chemistry, 221, 496–507. 27979233 10.1016/j.foodchem.2016.10.084

[fsn33320-bib-0038] Kandylis, P. , & Kokkinomagoulos, E. (2020). Food applications and potential health benefits of pomegranate and its derivatives. Food, 9(2), 122. 10.3390/foods9020122 PMC7074153 31979390

[fsn33320-bib-0039] Karimi, M. , Sadeghi, R. , & Kokini, J. (2017). Pomegranate as a promising opportunity in medicine and nanotechnology. Trends in Food Science and Technology, 69, 59–73.

[fsn33320-bib-0040] Kaur, P. , Ghoshal, G. , & Jain, A. (2019). Bio‐utilization of fruits and vegetables waste to produce β‐carotene in solid‐state fermentation: Characterization and antioxidant activity. Process Biochemistry, 76, 155–164.

[fsn33320-bib-0041] Kennas, A. , Amellal‐Chibane, H. , Kessal, F. , & Halladj, F. (2020). Effect of pomegranate peel and honey fortification on physicochemical, physical, microbiological and antioxidant properties of yoghurt powder. Journal of the Saudi Society of Agricultural Sciences, 19(1), 99–108.

[fsn33320-bib-0042] Khalil, A. A. , Khan, M. R. , Shabbir, M. A. , & Rahman, K. U. (2017). Comparison of antioxidative potential and punicalagin content of pomegranate peels. Journal of Animal and Plant Sciences, 27(2), 522–527.

[fsn33320-bib-0043] Khan, J. A. , & Hanee, S. (2011). Antibacterial properties of *Punica granatum* peels. International Journal of Applied Biology and Pharmaceutical Technology, 2, 23–27.

[fsn33320-bib-0044] Khan, S. , Patel, A. , & Bhise, K. S. (2017). Antioxidant activity of pomegranate peel powder. Journal of Drug Delivery and Therapeutics, 7(2), 81–84.

[fsn33320-bib-0045] Kharchoufi, S. , Licciardello, F. , Siracusa, L. , Muratore, G. , Hamdi, M. , & Restuccia, C. (2018). Antimicrobial and antioxidant features of 'Gabsiʼ pomegranate peel extracts. Industrial Crops and Products, 111, 345–352.

[fsn33320-bib-0046] Khedkar, R. D. , & Singh, K. (2014). New approaches for food industry waste utilization (pp. 15–12). Biologix, ISBN 81–88919.

[fsn33320-bib-0047] Ko, K. , Dadmohammadi, Y. , & Abbaspourrad, A. (2021). Nutritional and bioactive components of pomegranate waste used in food and cosmetic applications: A review. Food, 10(3), 657. 10.3390/foods10030657 PMC8003411 33808709

[fsn33320-bib-0049] Kumar, H. , Bhardwaj, K. , Sharma, R. , Nepovimova, E. , Kuča, K. , Dhanjal, D. S. , Verma, R. , Bhardwaj, P. , Sharma, S. , & Kumar, D. (2020). Fruit and vegetable peels: Utilization of high value horticultural waste in novel industrial applications. Molecules, 25(12), 2812. 32570836 10.3390/molecules25122812 PMC7356603

[fsn33320-bib-0050] Kumar, N. , Pratibha, N. , Petkoska, A. T. , Al‐Hilifi, S. A. , & Fawole, O. A. (2021). Effect of chitosan–pullulan composite edible coating functionalized with pomegranate Peel extract on the shelf life of mango (*Mangifera indica*). Coatings, 11(7), 764.

[fsn33320-bib-0051] Kumar, N. , Pratibha, P , A.T., Khojah, E. , Sami, R. , & Al‐Mushhin, A.A.M . (2021). Chitosan edible films enhanced with pomegranate Peel extract: Study on physical, biological, thermal, and barrier properties. Materials, 14(12): 3305. 34203852 10.3390/ma14123305 PMC8232757

[fsn33320-bib-0053] Kushwaha, S. C. , Bera, M. B. , & Kumar, P. (2013). Nutritional composition of detanninated and fresh pomegranate peel powder. IOSR Journal of Environmental Science, Toxicology and Food Technology, 7(1), 38–42.

[fsn33320-bib-0054] Lins, M. , Zandonadi, R. P. , Raposo, A. , & Ginani, V. C. (2021). Food waste on foodservice: An overview through the perspective of sustainable dimensions. Food, 10(6), 1175. 10.3390/foods10061175 PMC8225138 34073708

[fsn33320-bib-0055] Malviya, S. , Jha, A. , & Hettiarachchy, N. (2014). Antioxidant and antibacterial potential of pomegranate peel extracts. Journal of Food Science and Technology, 51(12), 4132–4137. 25477693 10.1007/s13197-013-0956-4 PMC4252460

[fsn33320-bib-0056] Manna, K. , Mishra, S. , Saha, M. , Mahapatra, S. , Saha, C. , Yenge, G. , Gaikwad, N. , Pal, R. , Oulkar, D. , Banerjee, K. , & Saha, K. D. (2019). Amelioration of diabetic nephropathy using pomegranate peel extract‐stabilized gold nanoparticles: Assessment of NF‐κB and Nrf2 signaling system. International Journal of Nanomedicine, 14, 1753–1777. 30880978 10.2147/IJN.S176013 PMC6413818

[fsn33320-bib-0057] Menezes, S. M. , Cordeiro, L. N. , & Viana, G. S. (2006). *Punica granatum* (pomegranate) extract is active against dental plaque. Journal of Herbal Pharmacotherapy, 6(2), 79–92. 17182487

[fsn33320-bib-0058] Middha, S. K. , Usha, T. , & Pande, V. (2013). A review on antihyperglycemic and Antihepatoprotective activity of eco‐Friendly Punica granatum Peel waste. Evidence‐Based Complementary and Alternative Medicine, 2013, 656172. 23878603 10.1155/2013/656172 PMC3708418

[fsn33320-bib-0059] Middha, S. K. , Usha, T. , & Pande, V. (2014). Pomegranate peel attenuates hyperglycemic effects of alloxan‐induced diabetic rats. EXCLI Journal, 13, 223–224. 26417256 PMC4464388

[fsn33320-bib-0060] Modaeinama, S. , Abasi, M. , Abbasi, M. M. , & Jahanban‐Esfahlan, R. (2015). Anti tumoral properties of *Punica granatum* (pomegranate) peel extract on different human cancer cells. Asian Pacific Journal of Cancer Prevention, 16(14), 5697–5701. 26320438 10.7314/apjcp.2015.16.14.5697

[fsn33320-bib-0061] Moghadam, M. , Salami, M. , Mohammadian, M. , Khodadadi, M. , & Emam‐Djomeh, Z. (2020). Development of antioxidant edible films based on mung bean protein enriched with pomegranate peel. Food Hydrocolloids, 104, 105735.

[fsn33320-bib-0062] Morzelle, M. C. , Salgado, J. M. , Massarioli, A. P. , Bachiega, P. , de Oliveira Rios, A. , Alencar, S. M. , Schwember, A. R. , & de Camargo, A. C. (2019). Potential benefits of phenolics from pomegranate pulp and peel in Alzheimer's disease: Antioxidant activity and inhibition of acetylcholinesterase. Journal of Food Bioactives, 5, 136–141.

[fsn33320-bib-0063] Morzelle, M. C. , Salgado, J. M. , Telles, M. , Mourelle, D. , Bachiega, P. , Buck, H. S. , & Viel, T. A. (2016). Neuroprotective effects of pomegranate peel extract after chronic infusion with amyloid‐β peptide in mice. PLoS One, 11(11), e0166123. 27829013 10.1371/journal.pone.0166123 PMC5102433

[fsn33320-bib-0064] Mphahlele, R. R. , Fawole, O. A. , Makunga, N. P. , & Opara, U. L. (2016). Effect of drying on the bioactive compounds, antioxidant, antibacterial and antityrosinase activities of pomegranate peel. BMC Complementary and Alternative Medicine, 16(1), 1–2. 27229852 10.1186/s12906-016-1132-y PMC4881059

[fsn33320-bib-0065] Mphahlele, R. R. , Fawole, O. A. , Makunga, N. P. , & Opara, U. L. (2017). Functional properties of pomegranate fruit parts: Influence of packaging systems and storage time. Journal of Food Measurement and Characterization, 11(4), 2233–2246.

[fsn33320-bib-0066] Mushtaq, M. , Gani, A. , Gani, A. , Punoo, H. A. , & Masoodi, F. A. (2018). Use of pomegranate peel extract incorporated zein film with improved properties for prolonged shelf life of fresh Himalayan cheese (Kalari/kradi). Innovative Food Science and Emerging Technologies, 48, 25–32.

[fsn33320-bib-0067] Omer, H. A. , Abdel‐Magid, S. S. , & Awadalla, I. M. (2019). Nutritional and chemical evaluation of dried pomegranate (*Punica granatum* L.) peels and studying the impact of level of inclusion in ration formulation on productive performance of growing Ossimi lambs. Bulletin of the National Research Centre, 43, 1–10.

[fsn33320-bib-0068] Pokorný, J. (2007). Are natural antioxidants better–and safer–than synthetic antioxidants? European Journal of Lipid Science and Technology, 109(6), 629–642.

[fsn33320-bib-0069] Prasad, D. , & Kunnaiah, R. (2014). *Punica granatum*: A review on its potential role in treating periodontal disease. Journal of Indian Society of Periodontology, 18(4), 428–432. 25210254 10.4103/0972-124X.138678 PMC4158581

[fsn33320-bib-0070] Rahnemoon, P. , Jamab, M. S. , Dakheli, M. J. , & Bostan, A. (2016). Phenolic compounds and antimicrobial properties of pomegranate (*Punica granatum*) peel extracts. International Journal of Agricultural and Biosystems Engineering, 10, 646–651.

[fsn33320-bib-0071] Sadq, S. M. , Ramzi, D. O. , Hamasalim, H. J. , & Ahmed, K. A. (2016). Growth performance and digestibility in Karadi lambs receiving different levels of pomegranate peels. Open Journal of Animal Sciences, 6(1), 16–23.

[fsn33320-bib-0072] Sagar, N. A. , Pareek, S. , Sharma, S. , Yahia, E. M. , & Lobo, M. G. (2018). Fruit and vegetable waste: Bioactive compounds, their extraction, and possible utilization. Comprehensive Reviews in Food Science and Food Safety, 17(3), 512–531. 33350136 10.1111/1541-4337.12330

[fsn33320-bib-0073] Saleh, H. , Golian, A. , Kermanshahi, H. , & Mirakzehi, M. T. (2017). Effects of dietary α‐tocopherol acetate, pomegranate peel, and pomegranate peel extract on phenolic content, fatty acid composition, and meat quality of broiler chickens. Journal of Applied Animal Research, 45(1), 629–636.

[fsn33320-bib-0074] Saroj, R. , Kushwaha, R. , Puranik, V. , & Kaur, D. (2020). Pomegranate peel: Nutritional values and its emerging potential for use in food systems. In Innovations in food technology (pp. 231–241). Springer.

[fsn33320-bib-0075] Shabbir, M. A. , Khan, M. R. , Saeed, M. , Pasha, I. , Khalil, A. A. , & Siraj, N. (2017). Punicic acid: A striking health substance to combat metabolic syndromes in humans. Lipids in Health and Disease, 16(1), 1–9. 28558700 10.1186/s12944-017-0489-3 PMC5450373

[fsn33320-bib-0076] Shabtay, A. , Eitam, H. , Tadmor, Y. , Orlov, A. , Meir, A. , Weinberg, P. , Weinberg, Z. G. , Chen, Y. , Brosh, A. , Izhaki, I. , & Kerem, Z. (2008). Nutritive and antioxidative potential of fresh and stored pomegranate industrial byproduct as a novel beef cattle feed. Journal of Agricultural and Food Chemistry, 56(21), 10063–10070. 18925742 10.1021/jf8016095

[fsn33320-bib-0077] Singh, B. , Singh, J. P. , Kaur, A. , & Singh, N. (2018). Phenolic compounds as beneficial phytochemicals in pomegranate (*Punica granatum* L.) peel: A review. Food Chemistry, 261, 75–86. 29739608 10.1016/j.foodchem.2018.04.039

[fsn33320-bib-0078] Singh, R. P. , Chidambara Murthy, K. N. , & Jayaprakasha, G. K. (2002). Studies on the antioxidant activity of pomegranate (*Punica granatum*) peel and seed extracts using in vitro models. Journal of Agricultural and Food Chemistry, 50(1), 81–86. 11754547 10.1021/jf010865b

[fsn33320-bib-0079] Smaoui, S. , Hlima, H. B. , Mtibaa, A. C. , Fourati, M. , Sellem, I. , Elhadef, K. , Ennouri, K. , & Mellouli, L. (2019). Pomegranate peel as phenolic compounds source: Advanced analytical strategies and practical use in meat products. Meat Science, 158, 107914. 31437671 10.1016/j.meatsci.2019.107914

[fsn33320-bib-0080] Smith, A. D. , George, N. S. , Cheung, L. , Bhagavathy, G. V. , Luthria, D. L. , John, K. M. , & Bhagwat, A. A. (2020). Pomegranate peel extract reduced colonic damage and bacterial translocation in a mouse model of infectious colitis induced by *Citrobacter rodentium* . Nutrition Research, 73, 27–37. 31841745 10.1016/j.nutres.2019.11.001

[fsn33320-bib-0081] Srivastava, P. , Indrani, D. , & Singh, R. P. (2014). Effect of dried pomegranate (*Punica granatum*) peel powder (DPPP) on textural, organoleptic and nutritional characteristics of biscuits. International Journal of Food Sciences and Nutrition, 65(7), 827–833. 25019979 10.3109/09637486.2014.937797

[fsn33320-bib-0082] Subash, S. , Essa, M. M. , Al‐Asmi, A. , Al‐Adawi, S. , Vaishnav, R. , Braidy, N. , Manivasagam, T. , & Guillemin, G. J. (2014). Pomegranate from Oman alleviates the brain oxidative damage in transgenic mouse model of Alzheimer's disease. Journal of Traditional and Complementary Medicine, 4(4), 232–238. 25379464 10.4103/2225-4110.139107 PMC4220500

[fsn33320-bib-0083] Tehranifar, A. , Selahvarzi, Y. , Kharrazi, M. , & Bakhsh, V. J. (2011). High potential of agro‐industrial byproducts of pomegranate (*Punica granatum* L.) as the powerful antifungal and antioxidant substances. Industrial Crops and Products, 34(3), 1523–1527.

[fsn33320-bib-0084] Topkaya, C. , & Isik, F. (2019). Effects of pomegranate peel supplementation on chemical, physical, and nutritional properties of muffin cakes. Journal of Food Processing and Preservation, 43(6), e13868.

[fsn33320-bib-0085] Ullah, N. , Ali, J. , Khan, A. , Khurram, M. , Hussain, A. , Rahman, I. U. , & Shafqatullah, Z. U. R. (2012). Proximate composition, minerals content, antibacterial and antifungal activity evaluation of pomegranate (*Punica granatum* L.) peels powder. Middle‐East Journal of Scientific Research, 11, 396–401.

[fsn33320-bib-0086] Umar, D. , Dilshad, B. , Farhan, M. , Ali, A. , & Baroudi, K. (2016). The effect of pomegranate mouthrinse on Streptococcus mutans count and salivary pH: An in vivo study. Journal of Advanced Pharmaceutical Technology and Research, 7(1), 13–16. 26955605 10.4103/2231-4040.173266 PMC4759979

[fsn33320-bib-0087] Vučić, V. , Grabež, M. , Trchounian, A. , & Arsić, A. (2019). Composition and potential health benefits of pomegranate: A review. Current Pharmaceutical Design, 25(16), 1817–1827. 31298147 10.2174/1381612825666190708183941

[fsn33320-bib-0088] Wang, Z. , Pan, Z. , Ma, H. , & Atungulu, G. G. (2011). Extract of phenolics from pomegranate peels. The Open Food Science Journal, 5, 17–25. 10.2174/1874256401105010017

[fsn33320-bib-0089] Yasoubi, P. , Barzegar, M. , Sahari, M. A. , & Azizi, M. H. (2007). Total phenolic contents and antioxidant activity of pomegranate (*Punica granatum* L.) peel extracts. Journal of Agricultural Science and Technology, 9, 35–42.

